# Off-label use of common predictive biomarkers in gastrointestinal malignancies: a critical appraisal

**DOI:** 10.1186/s13000-019-0843-z

**Published:** 2019-06-21

**Authors:** Basile Tessier-Cloutier, Ellen Cai, David F. Schaeffer

**Affiliations:** 1 Department of Pathology and Laboratory Medicine, University of British Columbia, Vancouver, BC Canada; 2 Department of Pathology and Laboratory Medicine, Vancouver General Hospital, 910 West 10th Ave, Vancouver, BC Canada

**Keywords:** Gastrointestinal tract cancer, HER2, MMR, PD-L1, BRAF V600E, ROS1, Immunohistochemistry, Predictive biomarkers

## Abstract

The use of immunohistochemistry (IHC) as a companion diagnostic is an increasingly important part of the case workup by pathologists and is often central to clinical decision making. New predictive molecular markers are constantly sought for to improve treatment stratification parallel to drug development. Unfortunately, official biomarker guidelines lag behind, and pathologists are often left hesitating when medical oncologists request off-labelled biomarker testing. We performed a literature review of five commonly requested off-label IHC predictive biomarkers in gastrointestinal tract (GIT) malignancies: HER2, mismatch repair (MMR), PD-L1, BRAF V600E and ROS1. We found that *HER2* amplification is rare and poorly associated to IHC overexpression in extracolonic and extragastric GIT cancers; however in *KRAS* wild type colorectal cancers, which fail conventional treatment, HER2 IHC may be useful and should be considered. For MMR testing, more evidence is needed to recommend reflex testing in GIT cancers for treatment purposes. MMR testing should not be discouraged in patients considered for second line checkpoint inhibitor therapy. With the exception of gastric tumors, PD-L1 IHC is a weak predictor of checkpoint inhibitor response in the GIT and should be replaced by MMR in this context. BRAF inhibitors showed activity in BRAF V600E mutated cholangiocarcinomas and pancreatic carcinomas in non-first line settings. *ROS1* translocation is extremely rare and poorly correlated to ROS1 IHC expression in the GIT; currently there is no role for ROS1 IHC testing in GIT cancers. Overall, the predictive biomarker literature has grown exponentially, and official guidelines need to be updated more regularly to support pathologists’ testing decisions.

## Introduction

The first immunohistochemistry (IHC) methods were developed in the 1930s, and as the technology evolved it quickly became a clinical standard in the 80s. Since then, there has been a vast increase in publication involving IHC techniques. Today, IHC is routinely used to support diagnosis and to guide treatment but as sequencing techniques become more available for directing personalized treatment, the overlap between the two technologies can cause confusion. Although many clinically useful genomic features can be detected using both IHC and DNA/RNA sequencing, clinical validation of genomic assays is still often lacking. Moreover, IHC instruments are ubiquitous among pathology laboratories and the cost of developing and running IHC assays is inexpensive and fast compared to the emerging next generation sequencing assays.

As part of the personalized medicine revolution, many cancers, including gastrointestinal malignancies, can be tested for biomarkers that are predictive of response to a targeted therapy. Current recommendations from the College of American Pathologists include HER2 IHC in esophageal and gastric adenocarcinomas, MMR IHC in colorectal and gastroesophageal adenocarcinomas and BRAF V600E IHC in colorectal adenocarcinomas only. [1–3] Results from basket and umbrella trials, comprehensive genomic analyses and even isolated case reports sometimes show benefit in off-label testing and treatment. [4, 5] Unfortunately guidelines take time to be updated and it is increasingly common for medical oncologists, sometimes pressured by their patients, to request off-label IHC testing.

**Fig. 1:**
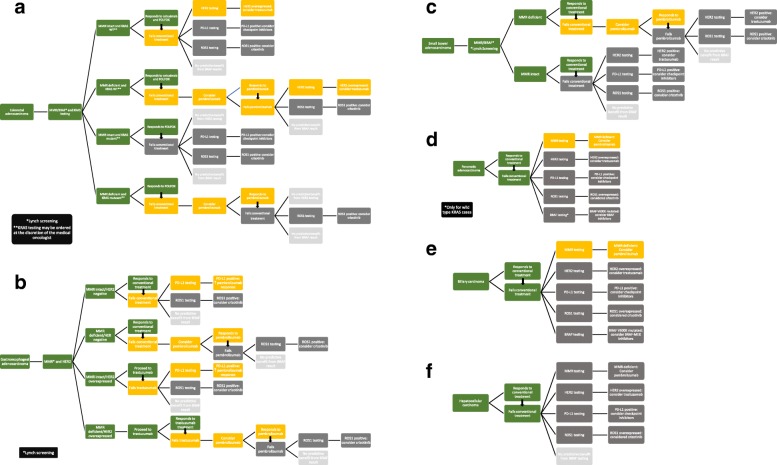
Evidence-based suggestions to aid decision making for off-label predictive and prognostic biomarkers in malignancies of the gastrointestinal tract. There are three degrees of evidence: high (green), intermediate (yellow), low (dark grey) and no evidence (pale grey). Based on tumor type: **a**) colorectal adenocarcinoma, **b**) gastroesophageal adenocarcinoma, **c**) small bowel adenocarcinoma, **d**) pancreatic adenocarcinoma, **e**) biliary adenocarcinoma and **f**) hepatocellular carcinoma

Since the surgical pathologist is responsible for the interpretation of IHC, the decision to perform a test not yet supported by clinical guidelines can be challenging. Even thorough review of the literature may not give clear direction, and the hesitation surrounding off-label testing is time consuming; therefore, there is possibility for misinterpretation. To aid decision making in situations where human epidermal growth factor receptor 2 (HER2), mismatch repair proteins (MMR), programmed death-ligand 1 (PD-L1), BRAF V600E and ROS1 IHC biomarkers are considered in gastrointestinal malignancies, we reviewed the literature and offer recommendations on an algorithmic approach to these biomarkers in the digestive tract. We created decision trees for each of the gastrointestinal sites to help visualize our conclusions by representing the degree of evidence as high (official management guidelines, in green), intermediate (clinical trials, in yellow), low (case series, case reports, retrospective reviews, in dark grey) and no evidence (in pale grey) (Fig. 1).

### HER2

The ERBB2/*HER2* gene, which encodes a transmembrane glycoprotein receptor, was at the center of the personalized medicine revolution in the early 2000s. When this gene is amplified, high levels of HER2 cell surface receptors quickly becomes the main driver for tumor progression. A recombinant monoclonal antibody, trastuzumab, was developed to recognize HER2 and trigger antibody-dependent cellular cytotoxicity; it inhibits HER2-mediated signaling, and prevents cleavage of the extracellular domain of HER2. [6, 7] In breast cancer and gastroesophageal cancers overexpressing HER2, trastuzumab has shown a survival advantage in early and metastatic disease and is now the standard of care. [1, 8]

Within GIT tumor types, HER2 expression is well described in colorectal and gastroesophageal adenocarcinomas; it is successfully targeted by HER2 specific tyrosine kinase inhibitors. [9, 10] With up to 5% amplification rate in colon cancer, the HERACLES trial showed high rates of response and prolonged disease control from HER2 targeted treatment in an otherwise treatment resistant group. This trial with other supporting evidence offer an explanation for potential mechanisms of resistance to HER2 kinase inhibitors, which is through *KRAS* inactivating mutations or other RAS pathway alterations. [9, 11, 12] Based on this evidence, we do not recommend HER2 testing in these *KRAS* mutated cases.

In other GIT sites, HER2 amplification is uncommon. Although one IHC and fluorescent in-situ hybridization (FISH) systematic review showed common overexpression and amplification with rates up to 28 and 23%, respectively, in hepatobiliary cancers such as ampullary carcinomas, extrahepatic biliary tract cancers, gallbladder carcinomas and extrahepatic cholangiocarcinomas, most other GIT cancers studies report overexpression rates below 10%. [13–21] In large scale next generation sequencing (NGS) data from the MSK-IMPACT trial, amplifications were rare: 0% in small bowel cancer (*n* = 35), 1.8% in pancreatic carcinomas (*n* = 502) and 3.4% in hepatobiliary cancers (*n* = 355). [22] It is unclear as to why the rates of amplification between FISH and NGS panels are so different, but race may be a factor since the FISH studies showing the high rates of amplification were based in Asia.

No clinical trials thus far have directly assessed the potential of HER2 inhibitors against HER2 amplified extracolonic/gastric GIT cancers. A cohort of patient-derived mouse xenograft benefited from cetuximab and trastuzumab over gemcitabine for the treatment of human pancreatic cancer; however, in a cohort of pancreatic cancer patients, a clinical trial assessing trastuzumab (not tested for *HER2* amplification) showed no significant response. [23, 24] There is one anecdotal report of a *HER2* amplified small bowel adenocarcinoma that displayed durable response to trastuzumab as second line therapy. [25] This finding is consistent with data in colorectal carcinoma (CRC) which exhibits activity from HER2 inhibitors. [9, 26]

There are no indications for HER2 IHC reflex testing in extracolonic and extragastric GIT carcinomas. The rates of 1–3% amplification could be worth investigating, especially for commonly unresponsive diseases like pancreatobiliary carcinomas, but the yield of large-scale reflex testing is unknown since no trials have shown benefit from HER2 inhibitors yet. Moreover, the relationship between *HER2* gene amplification and HER2 protein overexpression by IHC and their predictive potential are still unclear in these tumor types.

### Mismatch repair proteins

Mismatch repair (MMR) is an extremely well conserved mechanism across species that focusses on correcting DNA replication errors, especially in repetitive DNA sequences like microsatellites. MMR deficiency, defined by the loss of function in MSH2, MSH6, MLH1 or PMS2, typically causes microsatellite instability (MSI), which is associated with higher tumor antigenicity and response to programmed death 1 (PD-1) blockade. [4] MMR deficiency can result from a germline mutation (Lynch syndrome), a somatic mutation, or epigenetic promoter silencing. Alternatively, MSI can also be estimated using a polymerase chain reaction (PCR) based assay traditionally screening for events in 5 microsatellite loci. Depending on the number of regions showing alterations, a tumor is either define as MSI-high or MSI-low. [27]

In extracolonic malignancies, MMR deficiency is most common in small bowel adenocarcinomas (13–35%), hepatocellular carcinoma (22%) and pancreatic adenocarcinomas (15%). [13, 19, 28–33] In Barrett-esophagus associated adenocarcinomas, gastric carcinomas, and cholangiocarcinomas the loss of staining in MMR ranges from 0% to up to 10.9%, where it is generally also associated with longer survival. [28, 34–41]

In May 2017, for the first time in its history, the U.S. Food and Drug Administration approved a targeted therapy agnostic of site. Indeed, the results from Dung Le showed that MMR deficiency by IHC predicted response to pembrolizumab in 12 different malignancies including gastroesophageal, small bowel, pancreatobiliary and colorectal cancers, likely acting as a surrogate for high tumor mutational burden. [4, 42] Currently, any metastatic solid MMR deficient tumors which failed first line treatment can be considered for PD-1 blockade therapy. The study also observed that PD-L1 IHC status was not significantly associated with progression-free survival or overall survival.

In gastric cancer patients the MAGIC trial showed that MMR deficiency is associated with shorter survival when treated with surgery and adjuvant chemotherapy, but cases treated with surgery alone, MMR deficiency is associated with better prognosis. This trial was the first to suggest that loss of MMR function might be a predictor of poor response to perioperative epirubicin, cisplatin, and fluorouracil chemotherapy. [30]

Overall, MMR is now established as a predictive marker for response to pembrolizumab, with other checkpoint inhibitors are likely to follow. In this context testing for MMR should not be discouraged for metastatic tumors failing first line treatment. As discussed above MMR deficient tumors represent a significant portion of GIT cancers and accordingly, we can expect MMR IHC to be requested regularly in this demographic. Unlike some evidence that PD-L1 expression may increase following cytotoxic therapy, more research is needed to evaluate the effect of treatment on MMR and MSI status. [43] Until then we suggest testing metastatic tumor over primary tissue when available. It is also unclear if extracolonic cases showing MMR deficiency should be directly referred to a medical geneticist for Lynch syndrome work up, or if different guidelines should be applied since the association between MMR loss and Lynch syndrome is not as strong in these tumor types.

### PD-L1

With the emergence of immune checkpoint inhibitors, increasing interest has been placed on identifying a predictive biomarker for response. PD-L1 IHC was the first biomarker to show predictive potential in the initial checkpoint inhibitor trials. [44] This IHC targets the ligand, present on both tumor cells and lymphocytes, which interacts with the PD-1 receptor to suppress the immune response. [45] In lung adenocarcinomas PD-L1 IHC positivity is a pre-requisite for certain immune checkpoint inhibitor treatment, but for CRC, MMR status, acting as a surrogate for tumor mutational burden, is the preferred predictive biomarker as discussed above. [42, 46] Apart from its role in lung adenocarcinomas, PD-L1 is still under investigation as both a predictive and prognostic biomarker in other GIT malignancies with microsatellite stable profiles. [47]

PD-L1 IHC expression is common in GIT tumors and although it was often hypothesized to be a surrogate for high tumor mutational burden, similarly to MMR deficiency, a recent study on lung cancer suggest that they may in fact be uncorrelated. [48] The prognostic value of PD-L1 IHC expression is controversial. In previous studies, it did not show significant prognostic role in esophageal adenocarcinomas, but is favorable in squamous cell carcinoma of the esophagus. [49–51] Esophageal cancers showing epithelial-mesenchymal transition had higher PD-L1 expression. [50, 52] In gastric cancer it has been associated with microsatellite instability and a worse prognosis, whereas in pancreatic cancers, studies cannot reach consensus with regards to its prognostic prediction. [53] In both extrahepatic and intrahepatic biliary carcinomas, PD-L1 expression has been associated with worse prognosis. [54, 55] The mixed results of of the PD-L1 IHC across different tumor site could be explained by the different type of antibodies used, as well as patient selection.

In Keynote-059, PD-L1 IHC was used on a cohort of advanced gastric and gastroesophageal junction carcinomas prior to being treated with pembrolizumab. The expression recorded as positive if the combined positive score (ie. number of PD-L1–positive cells [tumor cells, macrophages, lymphocytes] divided by the total number of tumor cells, multiplied by 100) was greater than 1. In cohort 1 (pembrolizumab monotherapy as 3rd line), the PD-L1 positive patients’ (15.5%, 16.3 months) response rate and duration was over twice that of the PD-L1 negative group (6.4%, 6.9 months). [56] In cohort 2 (pembrolizumab-chemotherapy combination as first line), the observed response rate was higher in the PD-L1 positive group (68.8%) compared to the PD-L1 negative group (37.5%). [57] In other GIT cancers PD-L1 expression has not yet been prospectively tested as a predictive biomarker for checkpoint inhibitors. All GIT tumor, including CRC, tested in the initial pembrolizumab trial showed no benefit from therapy. [44] Further studies showed that tumor stratification based on PD-L1 expression or mutational load surrogate, improved the rate of response to checkpoint inhibitors but lacks sensitivity. [4, 58, 59] To date, this relationship in extracolonic GIT tumors remains mostly unexplored, let alone the percentage of tumor staining thresholds and the ideal antibody clone. Moreover, the amount of tumor tissue necessary to account for PD-L1 heterogenous expression needs to be established to consider its use in biopsy specimens.

### BRAF

Activating *BRAF* mutation, especially V600E, is a common mechanism for the constitutive activation of the MAP-kinase pathway, which has been observed in various malignancies. [60, 61] In metastatic melanomas, BRAF inhibitors have revolutionized treatment of many *BRAF* mutated tumors; however in CRC, BRAF inhibitors failed to show any benefits. [62, 63] BRAF V600E IHC is a readily available surrogate for the most common *BRAF* mutation, and is now evaluated as a predictive biomarker in other tumor types. [64, 65]

Multiple studies have investigated the incidence of *BRAF* V600E mutations in extracolonic gastrointestinal tumors and all showed dismal rates (< 1%). [66–76] The only extracolonic GIT sites that showed marginally higher frequency of *BRAF* mutations are cholangiocarcinomas (1.3%), and KRAS wild-type pancreatic adenocarcinomas (0–3%). [77–80] Case reports and now recently published preliminary results from the ROAR clinical trial show that metastatic *BRAF* mutant biliary tract cancers might benefit from BRAF and MEK inhibitors treatment. [81–83] As for pancreatic cancers, a recent case series of two metastatic *BRAF* mutated pancreatic adenocarcinomas (V600E and V600_601delinsG), suggested some benefit from BRAF inhibition. [84] We propose that off-label BRAF V600E testing can be performed for metastatic cholangiocarcinoma and KRAS wild type pancreatic adenocarcinomas in the non-first line setting. In other extracolonic GIT malignancies, currently there is no evidence for BRAF V600E IHC testing given the lack *BRAF* mutations and the lack of existing evidence for response to BRAF inhibition therapy.

### ROS1

*ROS1* is a known oncogene coding for a receptor tyrosine kinase that, similarly to the anaplastic lymphoma kinase (ALK), belongs to the insulin receptor superfamily. [85] The constitutive activation of *ROS1* from chromosomal rearrangements is well established in lung adenocarcinomas and is associated with durable response to crizotinib. [86, 87] The ROS1 IHC has shown good concordance with FISH and reverse transcription polymerase chain reaction (RT-PCR) assays in lung adenocarcinoma and is used routinely in some pathology laboratories as a predictive biomarker for crizotinib.

One study quoted 9% *ROS1* fusion from a phosphorylation assay using mass spectrometry, but NGS assays only found rare *ROS1* fusion in GIT cancers. [88] Based on data from The Cancer Genome Atlas (TCGA) and MSK-IMPACT trial, gastroesophageal cancers have a *ROS1* fusion rate of 2.6% (*n* = 341) and pancreatic carcinomas have a rate of 0.2% (*n* = 502). No fusion was identified in cholangiocarcinomas (*n* = 355) and CRC (*n* = 1007). [22, 89] The mass spectrometry results need to be confirmed using more conventional sequencing methods tailored to *ROS1* fusion detection in GIT malignancies.

The ROS1 IHC has shown good sensitivity and specificity as a surrogate marker for *ROS1* rearrangement in lung adenocarcinomas [90–95], but recent studies showed much lower specificity in gastric adenocarcinomas (3/23) and cholangiocarcinomas (0/72) when compared to NGS assays. [96, 97] So far there is no report of ROS1 inhibitors being used in cholangiocarcinomas and the potential use of ROS1 IHC expression for prognostic purposes is unclear. Two retrospective studies looking at survival based on ROS1 IHC showed opposing results, one suggested better prognosis and histologic differentiation and the other a worse prognosis. [97, 98] Tumors with low expression in ROS1, ALK, and c-MET IHC were associated with better response to a combination of gemcitabine, oxaliplatin and cetuximab, than those with high expression of ROS1, ALK and c-MET. [98]

Overall the data on *ROS1* in extracolonic cancers is sparse. Despite the increasing interest in targeted therapy in the rare cases with *ROS1* rearrangement, the poor concordance between IHC overexpression and the fusion supports no role for universal screening for *ROS1* alterations with ROS1 IHC in GIT malignancies.

### KRAS

*KRAS* mutation testing has shown to be an important predictive marker in colorectal carcinomas. [99–101] It is unclear if the decision to perform *KRAS* testing should be driven by the responsible pathologist or be requested by the treating medical oncologist. For the purpose of this review we excluded *KRAS* from the decision algorithms.

## Conclusion

In conclusion, the evolution of predictive biomarkers in diagnostic oncology is rapidly outpacing the established clinical guidelines, and pathologists will encounter increasing off-label testing requests. Substantial evidence supports screening for MMR deficiency in GIT adenocarcinomas and HER2 overexpression in colorectal adenocarcinomas when alternative treatment is needed. That said, there is an obvious need for evidence-driven testing algorithms and established roles for medical and diagnostic oncologists, so that predictive and prognostic biomarker testing can be optimized. Currently it is up to the pathologist to assess if a requested off-label test is appropriate based on his or her knowledge of the clinical context. In the absence of more regular guideline updates, review papers can be of great aid to the pathologist with evidence-based recommendations.

## Data Availability

N/A, no new patient data presented.
